# 4-Methylcatechol attenuates diabetic myocardial disorder via the ESR1–PI3K–AKT pathway

**DOI:** 10.3389/fphar.2026.1801465

**Published:** 2026-04-09

**Authors:** Zong-Hao Zhang, Ji-Fan Qian, Zehua Zhou, Jun-Xi Yin, Shi-Tao Zhou, Zhi-Hui Fang, Wei Qi, Hai-Yun Mao, Xiao-Jiang Wang, Rui Chen, Cheng-Zhi Fang, Hang Zhang, Hai-Hui Yang, Kai Hou

**Affiliations:** 1 Research Center, Pu’er People’s Hospital, Pu’er, Yunnan, China; 2 Research Center, Pu’er People’s Hospital, Kunming University of Science and Technology Affiliated Hospital, Pu’er, Yunnan, China; 3 Research Center, Pu’er People’s Hospital, School of Medicine, Kunming University of Science and Technology, Pu’er, Yunnan, China; 4 The Second Affiliated Hospital of Chengdu Medical College, China National Nuclear Corporation 416 Hospital, Chengdu, Sichuan, China; 5 Department of Radiology, Pu’er People’s Hospital, Pu’er, Yunnan, China; 6 Department of Cardiology, Tianjin Chest Hospital, Tianjin, China; 7 Department of Cardiology, Pu’er People’s Hospital, Pu’er, Yunnan, China; 8 Department of Traditional Chinese Medicine, Pu’er People’s Hospital, Pu’er, Yunnan, China; 9 School of Humanities and Management, Kunming Medical University, Kunming, Yunnan, China; 10 Department of Cardiothoracic Surgery, Pu’er People’s Hospital, Pu’er, Yunnan, China

**Keywords:** 4-methylcatechol, apoptosis, diabetic myocardial disorder, ESR1, mitochondria, oxidative stress, PI3K-Akt pathway

## Abstract

**Context:**

Diabetic myocardial disorder is a severe complication of diabetes mellitus, in which hyperglycemia and hyperlipidemia play pivotal roles in its pathogenesis.

**Objective:**

This study aimed to investigate the protective effects of 4-methylcatechol (4-MC) against diabetic myocardial injury and to elucidate its underlying mechanisms, using a high glucose and palmitic acid (HG/PA)-induced AC16 cardiomyocyte injury model. Materials and methods: To investigate 4-Methylcatechol (4-MC), potential therapeutic targets were first identified via bioinformatics, followed by molecular docking to analyze binding to the core target and KEGG/GO enrichment analyses to identify critical pathways, with final *in vitro* validation in AC16 cells assessing effects on HG/PA-induced oxidative stress and apoptosis, using pharmacological inhibitors to confirm the specific signaling axis.

**Results:**

Bioinformatics analysis identified ESR1 as a potential core therapeutic target of 4-MC. Molecular docking revealed that 4-MC forms stable hydrogen bonds with Arg394 and Lys449 residues within the ESR1 binding pocket. KEGG/GO enrichment analyses further indicated that the modulation of oxidative stress, apoptosis, and energy metabolism constitutes the critical pathways mediating 4-MC’s therapeutic effects. *In vitro* experiments demonstrated that 4-MC significantly mitigates HG/PA-induced oxidative stress and intrinsic apoptosis in cardiomyocytes. Notably, this cardioprotection was abolished upon treatment with the ESR1 antagonist Fulvestrant or the PI3K inhibitor LY294002, validating that 4-MC exerts its protective effect specifically through the ESR1-PI3K-AKT signaling axis.

**Conclusion:**

4-Methylcatechol alleviates diabetic myocardial disorder by activating the ESR1-PI3K-AKT pathway, offering novel therapeutic targets and a candidate compound for intervention.

## 1 Introduction

According to the International Diabetes Federation (IDF) 2024 statistics, 589 million adults (11.1%) worldwide have diabetes mellitus (DM), and this number is projected to increase to 852.5 million (13%) by 2050, highlighting the growing severity and global health burden of the disease (Ogle et al., 2025; Bielka et al., 2024). DM, characterized by chronic hyperglycemia and metabolic disturbances, leads to multiple systemic complications, among which cardiovascular diseases are particularly prominent (Yu et al., 2024; Accili et al., 2025). Diabetic myocardial disorder, as a specific cardiac manifestation of diabetes, represents a serious yet often underrecognized complication that deserves greater attention (Murtaza et al., 2019). The 2024 ESC consensus defines diabetic myocardial disorder as myocardial systolic and/or diastolic dysfunction associated with diabetes. The core pathophysiology involves metabolic disturbances—primarily lipotoxicity and glucotoxicity—driven by chronic hyperglycemia and insulin resistance (Seferović et al., 2024; Blüher, 2025). These disturbances contribute to oxidative stress, dysregulation of intracellular calcium handling, mitochondrial dysfunction (Lv et al., 2023), chronic inflammation, and progressive myocardial fibrosis (Lorenzo-Almorós et al., 2022). Morphologically, Diabetic myocardial disorder is characterized by cardiomyocyte hypertrophy and interstitial fibrosis, ultimately leading to impaired diastolic function and progression to heart failure (Scisciola et al., 2023). The pathogenic mechanisms of diabetic myocardial disorder remain largely unclear; however, accumulating evidence suggests roles for mitochondrial dysfunction, oxidative stress, cardiomyocyte apoptosis, and inflammation, highlighting the urgent need for further investigation to elucidate the underlying mechanisms and develop effective therapeutic strategies.

4-Methylcatechol (4-MC; also known as 3,4-dihydroxytoluene; CAS No. 452-86-8; molecular formula C_7_H_8_O_2_; molecular weight 124.14 g/mol) is a polyphenolic compound belonging to the catechol family (Hrubša et al., 2022), characterized by its ortho-dihydroxybenzene structure (Applová et al., 2019). It is an active metabolite of quercetin, a flavonoid abundantly present in various fruits including *apples*, *apricots*, *avocados*, *mangoes*, and *peaches* (Guo et al., 2021; Feliciano et al., 2016). Recent studies have indicated that 4-MC possesses antiplatelet and antihypertensive activities, contributing to a reduced incidence of cardiovascular diseases (Oudot et al., 2019). In terms of antioxidant effects, 4-MC has been shown to protect neural stem cells from oxidative stress-induced cell death (Draz et al., 2015). Molecular network analysis further reveals that 4-MC possesses potent antioxidant capacity (Aldian et al., 2025). Additionally, 4-MC has demonstrated anticancer activity in melanoma cells. Although these findings suggest diverse bioactivities of 4-MC, its potential role in diabetic myocardial disorder-a condition driven by oxidative stress, inflammation, and mitochondrial dysfunction-has not been explored. Moreover, the molecular target(s) through which 4-MC exerts its cardioprotective effects remain unknown. Although 4-MC has been demonstrated to possess cardioprotective, antioxidant, and anti-inflammatory effects, its mechanism of action remains unclear, and its therapeutic potential in the specific pathological context of diabetic myocardial disorder has not been systematically explored. Filling this gap is of great significance for understanding the bioactivities of 4-MC and its potential application in diabetic cardiomyopathy.

ESR1 encodes estrogen receptor-alpha (ERα), the primary estrogen receptor in humans. This protein serves as a critical component of the estrogen signaling pathway (Prossnitz and Barton, 2023). ESR1 is not only a key diagnostic biomarker for breast cancer treatment but also a major driver of breast cancer cell growth and survival (Jeselsohn et al., 2015). Beyond oncology, ERα activation exhibits cardioprotective effects in animal models, reducing infarct size, cardiomyocyte apoptosis, inflammation, and oxidative stress while promoting vasodilation and neovascularization (Puzianowska-Kuźnicka, 2012). Additionally, ERα plays an essential role in regulating mitochondrial morphology and function (Jeselsohn et al., 2015). Notably, ESR1 has been identified as a potential therapeutic target for various natural compounds in treating heart failure (Chen et al., 2024).

In this study, we aim to fill this gap by systematically investigating the interaction between 4-MC and ESR1 using integrated computational and experimental approaches. We first performed molecular docking to screen potential targets of 4-MC and identified ESR1 as a top candidate. Molecular dynamics simulations were then employed to characterize the binding mode and stability of the 4-MC-ESR1 complex. Subsequently, pharmacological blockade with the ESR1-specific antagonist Fulvestrant was used to validate the functional dependence of 4-MC’s cardioprotective effects on ESR1 in AC16 cardiomyocytes under diabetic conditions. This study reveals a direct interaction between 4-MC and ESR1 and elucidates the molecular basis of this interaction in the context of diabetic myocardial disorder, thereby providing a novel perspective on the mechanism of action of 4-MC. Our findings not only uncover a novel mechanism of action for 4-MC but also position ESR1 as a key mediator of its cardioprotective effects, providing a rationale for developing 4-MC-based therapeutic strategies against diabetic cardiomyopathy.

## 2 Materials and methods

### 2.1 Sources of 4-methylcatechol

4-Methylcatechol (CAS No. 452-86-8; Cat. No. HY-W012814) was purchased from MedChemExpress (MCE). The compound was dissolved in DMSO for *in vitro* experiments.

### 2.2 Cell culture

AC16 cells were purchased from Servicebio (AC16,Human hybrid cardiomyocyte, RRID:CVCL-4U18, catalogue numbers: STCC13101P), China cultured in Dulbecco’s modified Eagle’s medium (DMEM; G4512-500 mL,Servicebio) supplemented with 10% fetal bovine serum (FBS; G8003-100 mL,Servicebio) and 1% penicillin/streptomycin (15140122, Gibco, Thermo Fisher). Cells were maintained at 37 °C in a 5% CO_2_ humidified incubator. AC16 cells were treated with high glucose (HG, 30 mM) and palmitic acid (PA, 200 μM) to mimic diabetic myocardial disorder. Cells were harvested at 0,12,24,48and 96 h post-treatment.4-Methylcatechol (HY-W012814) was purchased from MedChemExpress (MCE).

### 2.3 CCK8 assays

AC16 cells were seeded in 96-well plates at a density of 3 × 10^4^ cells/mL and cultured at 37 °C. After treatment, cells were washed twice with PBS before incubation with 10 μL CCK-8 reagent (MA0218, MeilunBio, Dalian, China) in 90 μL DMEM for 2 h at 37 °C. Absorbance at 450 nm was determined using a microplate reader (Thermo Fisher, United States) to calculate optical density (OD).

### 2.4 Collection of 4-MC targets

First, we retrieved the PubChem CID and the standardized SMILES for 4-MC from the PubChem database (https://pubchem.ncbi.nlm.nih.gov/) to ensure consistent and reusable chemical representations. We then queried the BATMAN-TCM 2 (Xiangren et al., 2023). platform with the CID to predict candidate targets, applying a stringent score cutoff of 0.6 (likelihood ratio, LR = 13.26 (Zhongyang et al., 2013)) to increase confidence in the results. In parallel, we used the SMILES in SwissTargetPrediction (David et al., 2014), restricted the species to *Homo sapiens*, and retained targets with probability >0 to maximize coverage of potentially relevant proteins. Finally, we merged and deduplicated outputs from both platforms to assemble a multi-evidence set of 4-MC-related genes, providing a high-quality basis for subsequent functional enrichment, pathway analysis, and mechanistic studies.

#### 2.4.1 Collection of DCM/diabetic myocardial disorder -related targets

In this study, we initially obtained protein annotation data from the UniProt database (https://www.uniprot.org/) filtered by “Reviewed” and “Human” entries. Subsequently, we queried PubMed via NCBI E-utilities API using search terms related to diabetic cardiomyopathy and diabetic myocardial disorder. Building upon this, we conducted advanced searches in Web of Science, exported the retrieved records to Excel, and ultimately integrated and deduplicated the results from both PubMed and Web of Science to generate a curated dataset of diabetic myocardial disorder-associated genes. The entire workflow was implemented in Python 3.13 with core dependencies including requests, lxml/BeautifulSoup, re, pandas, seaborn, and matplotlib.

#### 2.4.2 Calculation of intersecting targets

We used R 4.2.1 with the ggplot2 and VennDiagram packages to compute the intersection between DCM-related genes and the predicted targets of 4-MC, and visualized the overlap using a Venn diagram.

#### 2.4.3 PPI analysis

We queried the STRING database with the intersecting gene set to perform protein–protein interaction (PPI) analysis, restricting the species to *H. sapiens* and applying the highest-confidence threshold (combined score ≥0.9). The resulting PPI network was imported into Cytoscape 3.7.0, and the top 10 hub genes were identified using the cytoHubba plugin based on degree centrality.

#### 2.4.4 GO enrichment and KEGG analysis

We used R 4.2.1 and the clusterProfiler package to convert identifiers for the intersecting targets shared by diabetic cardiomyopathy (DCM)/diabetic myocardial disorder and 4-MC, followed by GO enrichment and KEGG analyses with clusterProfiler. The organism was set to *H. sapiens*, and identifier mapping was performed with the org. Hs.e.g. db annotation package.

### 2.5 Molecular docking and molecular dynamics simulations

#### 2.5.1 Molecular docking

We retrieved the SDF file for 4-MC from PubChem (https://pubchem.ncbi.nlm.nih.gov) and the PDB file for the target protein from the RCSB PDB (https://www.rcsb.org). The small-molecule ligand was energy-minimized with the MM2 force field in ChemBio3D 14.0. Protein preprocessing in PyMOL 3.1 involved removing nonessential water molecules and bound organic ligands. Hydrogen atoms and partial charges were added to both protein and ligand using AutoDockTools 1.5.7, and molecular docking was performed with AutoDock Vina.

#### 2.5.2 Molecular dynamics

We initiated molecular dynamics (MD) simulations from docking-derived complexes to evaluate the stability of binding poses and intermolecular interactions. First, the receptor and ligand were extracted from AutoDock-generated PDBQT complex files, separated and exported in PyMOL, and the protein structure was repaired using Swiss-PdbViewer 4.10. System topologies were then generated in GROMACS 2023.2, applying the AMBER99SB force field to the protein and GAFF to the ligand. The system was solvated in an orthorhombic box with TIP3P water and neutralized with counterions. Energy minimization employed steepest-descent followed by conjugate-gradient algorithms (nsteps = 10,000). Equilibration proceeded in two phases (NVT then NPT) at a 2fs time step, continuing until temperature and pressure stabilized. Production MD was conducted for 100 ns with a 2fs time step (nsteps = 50,000,000). After the simulations, trajectories were de-drifted and aligned; RMSD, SASA, radius of gyration, and hydrogen bonds were computed; and a free-energy landscape was constructed. Finally, binding free energy was estimated with gmx-mmpbsa (Mario S et al., 2021), and total and per-residue free-energy contribution plots were generated.

### 2.6 In-Cell Western assays

AC16 cells were seeded in 96-well plates at a density of 5,000 cells per well and cultured at 37 °C for 24 h prior to drug treatment. After removing the culture medium, cells were fixed with 150 μL ice-cold methanol (Saifurui Chemical Handan Co, cat#67-56-1) at 4 °C for 20 min, followed by permeabilization with 150 μL 0.1% Triton X-100 (Biofroxx, cat#9002-93-1) at room temperature for 30 min. Subsequent to permeabilization, the solution was aspirated, and blocking was performed using 50 μL LI-COR blocking buffer (NC1660550) at room temperature for 1.5 h. Without further washing, 50 μL diluted primary antibody (1:50–1:500) was added, and plates were incubated overnight at 4 °C. Following washing steps, cells were incubated with 50 μL secondary antibody (1:800 dilution) shielded from light at room temperature for 1 h. After a final wash with PBS, quantification was performed using an Odyssey DLX infrared imaging system (LICOR) at a focal depth of 4.0 mm.

### 2.7 Western assays

Cells were washed twice with PBS, resuspended in cold PBS, and centrifuged at 12,000 rpm for 10 min at 4 °C. Pellets were lysed in RIPA buffer containing protease/phosphatase inhibitors (ice, 20 min). Lysates were centrifuged (12,000 rpm, 4 °C, 10 min), and supernatants were quantified by BCA assay (#P0012, Beyotime). Equal protein amounts were separated by 12% SDS-PAGE and transferred to NC membranes. Membranes were blocked with 5% milk (2 h, RT), then incubated with primary antibodies (P-AKT, AKT, P-PI3K, PI3K, GAPDH, Bcl-2, Bax, cleaved caspase-3; 4 °C, overnight). After PBST washes, membranes were incubated with HRP-conjugated secondary antibodies (1–2 h, RT).

### 2.8 Immunofluorescence staining

AC16 cells were washed three times with cold PBS, fixed with 4% paraformaldehyde (BL539A, biosharp) for 10 min at 37 °C, and permeabilized with 0.1% Triton X-100 (#9002-93-1, Biofroxx) for 15 min at room temperature. Subsequently, cells were blocked with 5% BSA (A66421-100 g, ACMEC) for 30 min, followed by incubation with specified primary and secondary antibodies at 4 °C for 12 h. Fluorescent imaging was performed using a laser scanning confocal microscope (BZ-X810, KEYENCE), and quantitative fluorescence intensity/colocalization analysis was conducted with ImageJ software (v2.1.4.8, NIH, United States).

### 2.9 TUNEL assays

Apoptosis was assessed using a TUNEL assay kit (#C1089, Beyotime) following the manufacturer’s instructions. TUNEL-positive cells were visualized and quantified under a confocal microscope (KEYENCE, BZ-X810).

## 3 Results

### 3.1 4-MC alleviated the injury induced by combined HG and PA treatment in AC16 cardiomyocytes

**FIGURE 1:**
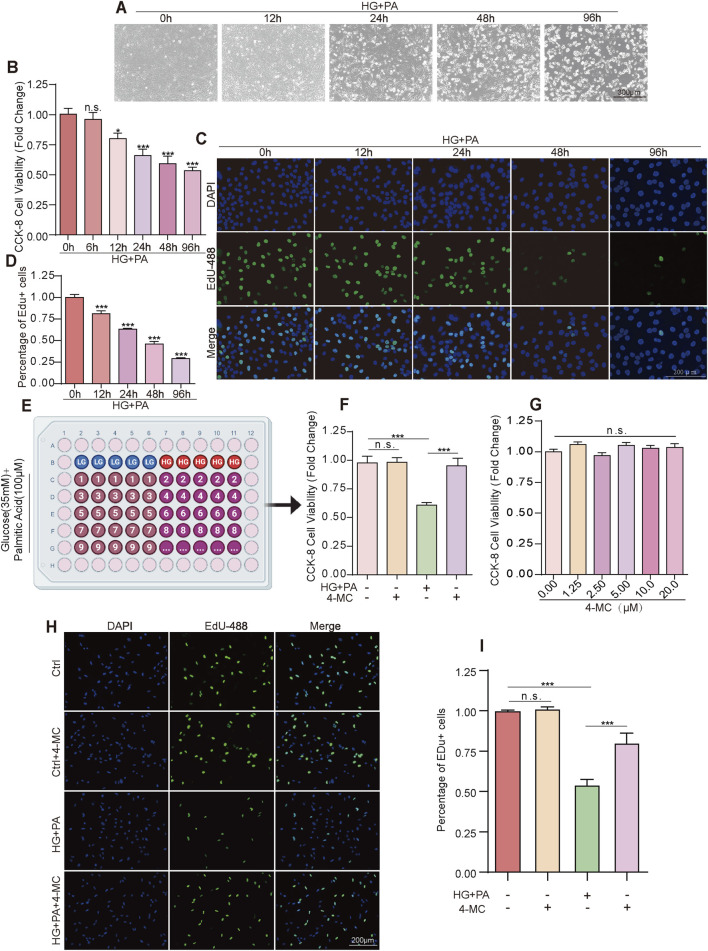
4-MC Maintains Cell Viability and Suppresses HG + PA-Induced Cytotoxicity in AC16 Cells. **(A)** Morphological changes of AC16 cells with treatment time. **(B)** The CCK-8 assay determined the viability of AC16 cells treated with HG + PA across a time gradient. **(C,D)** EdU assay assesses the proliferation of AC16 cells treated with HG + PA over a time gradient. **(E)** Monomeric Compound Library of Traditional Chinese Medicine. **(F)** 4-MC alleviates HG/PA-induced cytotoxicity in AC16 cells. **(G)** CCK-8 assay results for control cells treated with varying concentrations of 4-MC. **(H,I)** 4-MC restores HG/PA-inhibited proliferation in AC16 cells. The results represent at least three independent experiments; data are presented as the mean ± SD (n ≥ 3) n. s., non-significant, *p < 0.05, **p < 0.01, and ***p < 0.001.

To establish an *in vitro* model of diabetic myocardial disorder, the immortalized human cardiomyocyte cell line AC16 was incubated in a medium containing HG (35 mM) and PA (100 μM) to mimic the diabetic conditions. Cells maintained in low glucose (LG, 4.5 mM) medium served as the control. AC16 cells treated with the combination of HG and PA exhibited significant time-dependent morphological characteristics of cell death, observed at 12, 24, 48, and 96 h post-treatment (Figure 1A). The CCK8 assay demonstrated that cell viability gradually decreased with prolonged treatment time under combined HG and PA treatment (Figure 1B). Furthermore, this inhibitory effect was corroborated by EdU (5-Ethynyl-2′-deoxyuridine) staining, which confirmed that combined HG and PA treatment significantly suppressed AC16 cell proliferation (Figures 1C,D). From a library of 2,800 natural compounds, we identified 4-MC as a hit compound capable of significantly restoring the cell viability suppressed by combined HG and PA treatment (Figures 1E,F). However, 4-MC had no significant effect on the viability of AC16 cells under LG culture conditions (Figure 1G). Furthermore, 4-MC effectively alleviated the inhibition of cell proliferation induced by combined HG and PA treatment. (Figures 1H,I). Based on the protective effect of 5 μM 4-MC on cardiomyocytes, subsequent experiments were conducted using 4-MC at this concentration.

### 3.2 Target prediction and network pharmacology analysis of 4-MC

**FIGURE 2:**
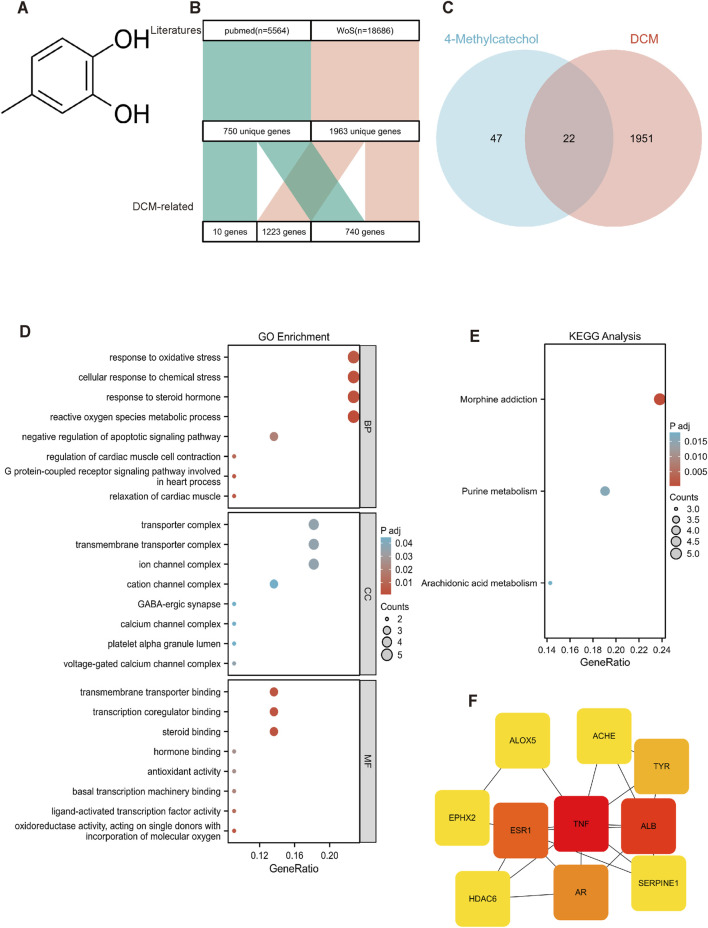
Network pharmacology analysis clarify the possible mechanisms of 4-MC and diabetic myocardial disorder activity. **(A)** Structures of 4-MC. **(B)** Flowchart of published studies and target screening validation. **(C)** Venn diagram of common 4-MC and diabetic myocardial disorder/DCM targets. **(D,E)** KEGG and GO analysis results of potential targets. **(F)** Venn diagram of common targets between 4-MC and diabetic myocardial disorder.

To explore the protective mechanism of 4-MC (structure shown in Figure 2A) against combined HG and PA treatment-induced cardiomyocyte injury, we constructed a set of diabetic myocardial disorder-related targets. An initial literature search of PubMed and Web of Science retrieved 24,250 publications related to diabetic myocardial disorder (Figure 2B). From these, we selected 1,951 frequently reported target genes highly associated with diabetic myocardial disorder and successfully established a diabetic myocardial disorder-related target database. Subsequently, based on databases including TCMSP, GeneCards, SwissTargetPrediction (STP), PharmMapper (PM), and BindingDB, we systematically predicted and identified 48 potential targets of 4-MC. The intersection of the diabetic myocardial disorder-related and 4-MC target datasets via Venn analysis yielded 27 shared targets. From these, molecular docking identified the top 10 core targets as hub genes, for which a protein-protein interaction network was constructed (Figure 2F). Functional enrichment analysis (GO/KEGG) indicated that 4-MC is involved in biological processes related to oxidative stress response, regulation of cardiac muscle contraction, and modulation of cardiac function. The associated genes were mainly localized to membrane complexes such as transporters and ion channels and exhibited molecular functions associated with oxidative stress response and metabolic regulation. (Figures 2D,E). Taken together, the analysis implicates the amelioration of oxidative stress and the restoration of energy metabolic homeostasis as core mechanisms through which 4-MC confers protection in diabetic myocardial disorder.

### 3.3 Molecular docking and molecular dynamics simulations of 4-MC

**FIGURE 3:**
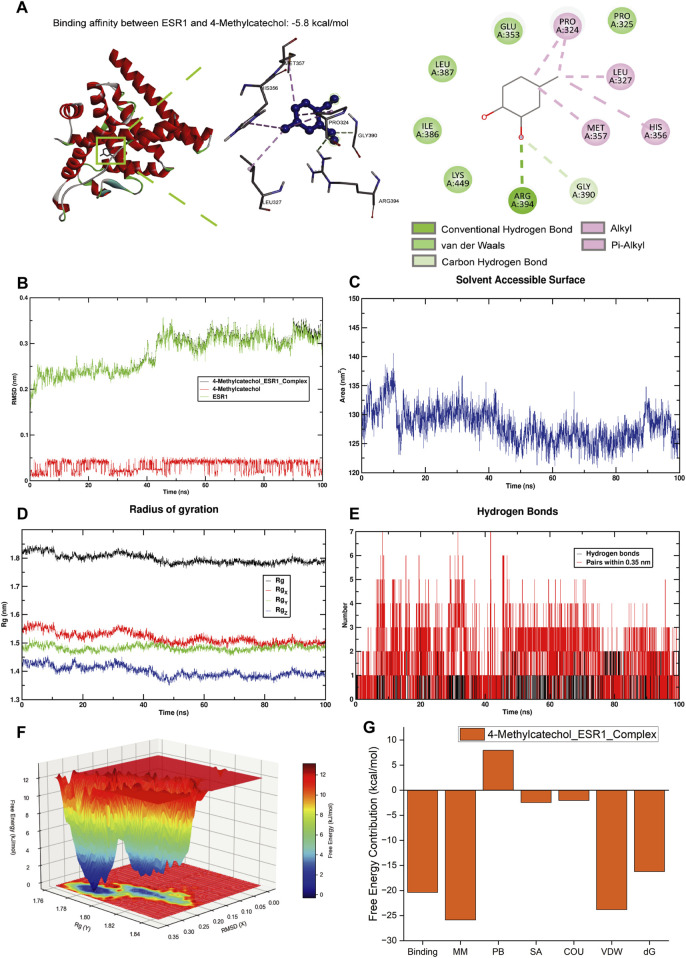
Integrated molecular docking and molecular dynamics simulations identified 4-MC as a putative target of ESR1. **(A)** 3D and 2D interaction diagram of 4-MC in ESR1 binding sites. **(B)** RMSD of the protein-ligand complex (com). **(C)** solvent-accessible surface area (SASA) plot. **(D)** Radius of gyration (Rg) plot. **(E)** Number of hydrogen bond interactions during the evolution of the simulation. **(F)** The free energy landscape (FEL) reveals the conformational changes of ESR1 in complex with 4-MC. **(G)** The MM/PBSA energy analysis of 4-MC binding to ESR1 yielded a total binding free energy of −20 kcal/mol.

We performed molecular docking for seven genes among the top 10 intersecting targets of 4-MC and ESR1, AR, EPHX2, ALOX5, HDAC6, ACHE, SERPINE1 (Figure 3A; Supplementary Figure S1). Docking results suggested that 4 MC forms stable complexes with multiple candidate targets. We selected the ESR1-4-MC complex for molecular dynamics simulations because it exhibited a well defined interaction pattern-hydrogen bond anchoring with Arg394 and Lys449 together with a stable docking score (−5.8 kcal/mol). Importantly, ESR1 confers cardioprotection by modulating SIRT3, CSRP3 (Chen et al., 2024), and PI3K-AKT-NF-κB signaling (Chen et al., 2025), and is implicated as a therapeutic target for flavonoids like isorhamnetin and quercetin in heart failure (Cai et al., 2025).

We conducted a 100-ns molecular dynamics simulation to evaluate the binding stability of the 4-MC-ESR1 complex. As shown in Figure 3B, the conformational fluctuations of the complex closely mirrored those of the ESR1 protein itself, indicating that the ligand maintained stable spatial positioning within the binding pocket. The solvent-accessible surface area (SASA) remained relatively constant throughout the simulation, reflecting a stable conformational state at the binding interface (Figure 3C). The radius of gyration (Rg) of the complex decreased gradually during the mid-to-late stages of the simulation and eventually plateaued, suggesting that the system progressively reached equilibrium (Figure 3D). Interaction analysis revealed that the binding between 4-MC and ESR1 was primarily driven by hydrophobic interactions and van der Waals forces, supplemented by a limited number of dynamic hydrogen bonds, without the formation of strong or persistent polar interaction networks (Figure 3E). The positional fluctuation of 4-MC within the binding cavity was minimal, demonstrating excellent shape complementarity with the pocket. The free energy landscape showed that the system predominantly populated a single low-energy state, characterized by a root-mean-square deviation (RMSD) of approximately 0.05–0.20 nm and a radius of gyration between 1.78 and 1.81 nm, further confirming the stability of the binding (Figure 3F). The binding pocket is predominantly composed of hydrophobic residues, with shape complementarity and van der Waals interactions identified as the key determinants of binding (Figure 3G). These data support a stable, hydrophobically driven association between 4-MC and ESR1.

### 3.4 4-MC alleviates HG/PA-triggered oxidative stress and mitochondrial-mediated apoptosis in cardiomyocytes

**FIGURE 4:**
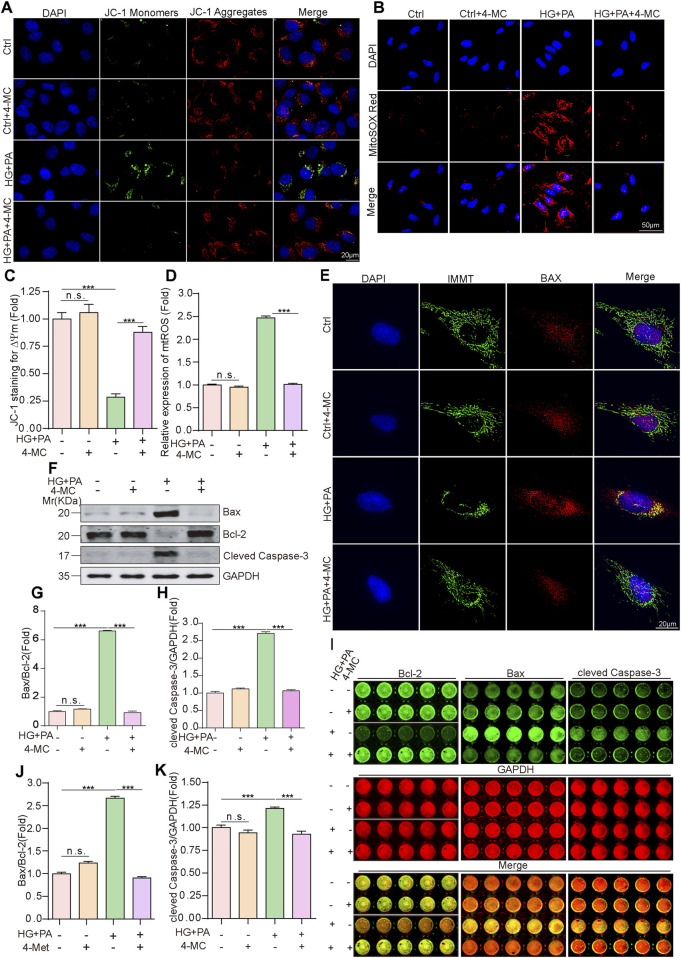
4-MC attenuates HG + PA-induced oxidative stress and apoptosis in AC16 cells. **(A,C)** JC-1 staining revealed a bicolor distribution of mitochondrial membrane potential in AC16 cells. Scale bar:20 μm. **(B,D)** MitoSOX Red fluorescence staining revealed the accumulation of mitochondrial superoxide in AC16 cells. Scale bar:50 μm. **(E)** Immunofluorescence was carried out using a mitochondrial AIF antibody (IMMT, green) and a conformation-specific anti-BAX antibody (clone 6A7, red). Scale bar:20 μm. **(F–H)** Western blotting was performed to assess the protein levels of BAX, Bcl-2 and cleaved caspase-3 across different experimental groups. **(I–K)** In-cell Western blotting was performed to assess the protein levels of BAX, Bcl-2 and cleaved caspase-3 across different experimental groups. Data shown are mean ± standard deviation. Student’s t-test was used to compare the results. n. s., nonsignificant; ***p < 0.001.

To determine whether the protective effect of 4-MC against combined HG and PA treatment-induced myocardial injury is mediated through the pathways of cellular oxidative stress and apoptosis, JC-1 (5,5′,6,6′-tetrachloro-1,1′,3,3′-tetraethylbenzimidazol-carbocyanine iodide) was used to detect mitochondrial membrane potential (ΔΨm). The combined HG and PA treatment exhibited a significant decrease in the JC-1 red/green fluorescence intensity ratio, indicating HG/PA-induced mitochondrial injury, whereas 4-MC treatment markedly attenuated this reduction (Figures 4A,C). Analysis of MitoSOX Red staining revealed significant accumulation of mitochondrial superoxide in the combined HG and PA treatment group, which was effectively reversed by 4-MC treatment (Figures 4B,D). This suggests that under HG and PA co-treatment condition, the loss of mitochondrial membrane potential leads to oxidative stress and substantial ROS accumulation, and this phenotype can be restored by 4-MC. Given that excessive ROS often triggers mitochondrial apoptosis, we performed co-localization analysis using MitoTracker Green and the pro-apoptotic protein Bax. In the HG and PA co-treatment group, mitochondria exhibited fragmented punctate structures accompanied by the accumulation of Bax on the outer mitochondrial membrane. In contrast, both the control and 4-MC treatment groups exhibited a reticular mitochondrial network with minimal Bax co-localization (Figure 4E). These findings suggest increased mitochondrial outer membrane permeability and the subsequent release of cytochrome c (Cyt c), thereby activates the intrinsic apoptotic pathway. Further assessment using Western blot and In-Cell Western assays revealed that the combined HG and PA treatment markedly increased levels of key apoptosis-related markers, including the Bax,Bcl-2 and cleaved Caspase-3. In contrast, 4-MC treatment significantly reversed HG + PA co-treatment-induced alterations in apoptotic markers. Specifically, 4-MC reduced HG + PA-stimulated expression levels of Bax (Supplementary Figure S2C) and cleaved caspase-3 (Figure 4F) while decreasing the Bax/Bcl-2 ration (Figure 4G; Supplementary Figure S2D). TUNEL staining results confirmed that 4-MC treatment ameliorated the combined HG and PA treatment-induced apoptosis (Supplementary Figure S3A,B). Together, these results indicsate that 4-MC alleviates HG + PA-induced oxidative stress and intrinsic apoptosis.

### 3.5 4-MC alleviates oxidative stress by activating ESR1 and restoring the activity of the PI3K-Akt signaling pathway

**FIGURE 5:**
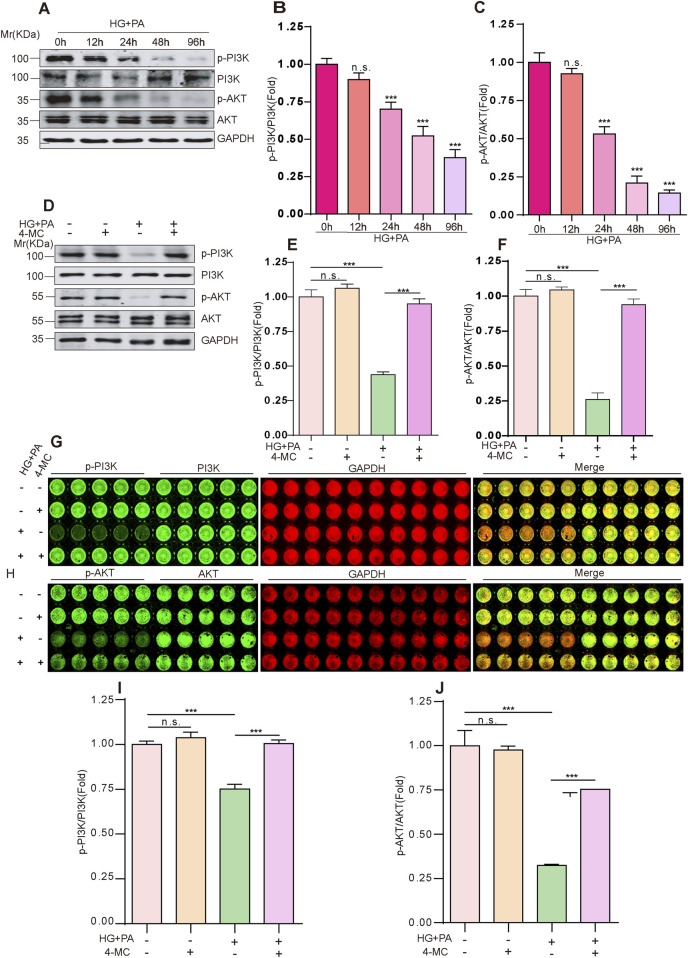
4-MC Maintains Cell Viability and Suppresses HG + PA-Induced Cytotoxicity in AC16 Cells. **(A–C)** AC16 cells were treated with HG + PA for 12, 24, 48, and 96 h, followed by evaluation of PI3K and Akt phosphorylation levels via Western blotting. **(D,E)** 4-MC activated PI3K and Akt phosphorylation that was suppressed by HG + PA in AC16 cells. **(F–J)** In-cell Western blotting was performed to quantify PI3K and Akt phosphorylation levels in AC16 cells under HG + PA and 4-MC conditions. Densitometry of immunoblot bands was determined by the Odyssey Infrared Imaging System. Data shown are mean ± standard deviation. Student’s t-test was used to compare the results. n. s., nonsignificant; ***p.

The estrogen receptor ESR1, a member of the nuclear receptor superfamily, promotes cell survival and proliferation via pathways such as PI3K-AKT/MAPK/ERK and G protein-coupled receptors (GPCR) (Liu et al., 2023),with the PI3K-AKT axis being its primary route for regulating apoptosis (Jiang et al., 2025) and myocardial infarction (Chen et al., 2025; Liu et al., 2025), PI3K-AKT axis is also one of the critical pathways modulating systemic glucose homeostasis (Ma et al., 2025; Ghigo and Li, 2015). Western blot analysis revealed a time-dependent decrease in the phosphorylation levels of PI3K (P85α/P55γ/P85β-Y467/Y464/Y199) and AKT (Thr308) under the combined HG and PA treatment (Figures 5A,B). The suppression of PI3K and AKT phosphorylation was reversed by 4-MC treatment (Figures 5C–E), In-Cell Western produced concordant results (Figures 5F–I).

**FIGURE 6:**
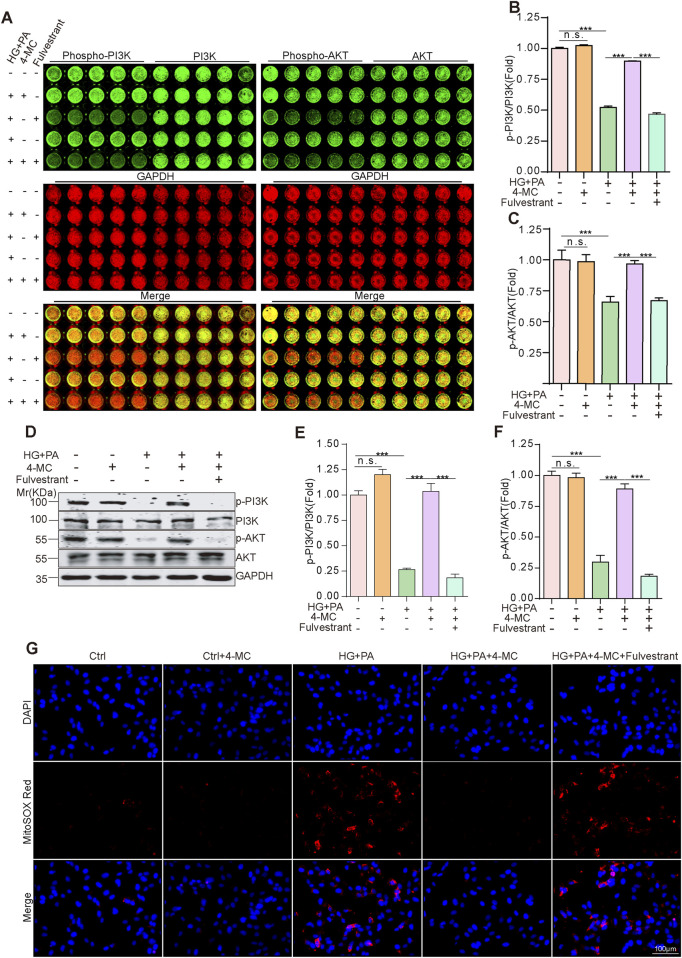
4-MC activates ESR1 to rescue HG + PA-suppressed PI3K/AKT signaling and alleviate oxidative stress in AC16 cells. **(A–C)** In-cell WB analysis of PI3K/Akt phosphorylation (phospho/total ratios). **(D–F)** Western blotting analysis of PI3K/AKT phosphorylation (phospho/total ratios). **(G)** MitoSOX Red fluorescence staining revealed the accumulation of mitochondrial superoxide in AC16 cells. Scale bar:100 μm. Data shown are mean ± standard deviation. Student’s t-test was used to compare the results. n. s., non-significant; ***p.

To elucidate whether 4-MC activates the PI3K-AKT signaling pathway through ESR1, we employed fulvestrant, a selective ESR1 degrader, to specifically induce ESR1 deficiency (Kingston et al., 2024). Following fulvestrant treatment, 4-MC lost its ability to reverse the inhibition of PI3K and AKT phosphorylation induced by combined HG and PA treatment, and this phenomenon was likewise confirmed by In-Cell Western (Figures 6A–C). In addition, the JC-1 assay results also showed that fulvestrant blocked the effect of 4-MC in reversing the oxidative stress induced by combined HG and PA treatment (Figure 6E; Supplementary Figure S3K). Taken together, 4-MC restores the activity of the PI3K-AKT signaling pathway, which is suppressed by the combined HG and PA treatment, by activating ESR1, and ESR1 is essential for the ability of 4-MC to alleviate oxidative stress induced by the combined HG and PA treatment.

### 3.6 4-MC alleviates diabetic cardiomyocyte injury by activating the PI3K-AKT signaling pathway

**FIGURE 7:**
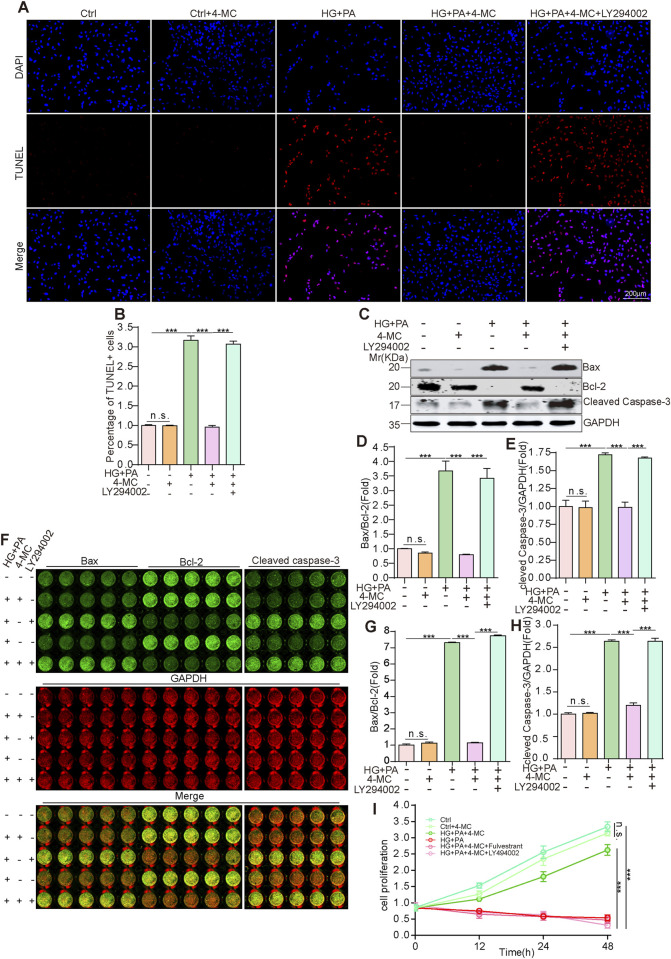
4-MC suppresses HG + PA-induced oxidative stress and apoptosis via PI3K/AKT pathway. **(A,B)** TUNEL assay detects apoptosis in AC16 cells. Scale bar: 200 μm. **(C–E)** Western blotting was performed to assess the protein levels of BAX, Bcl-2 and cleaved caspase-3 across different experimental groups. **(F -H)** In-cell Western blotting was performed to assess the protein levels of BAX, Bcl-2 and cleaved caspase-3 across different experimental groups. **(I)** The viability of cells in each group was directly determined using the CCK-8 assay, with raw absorbance values reported. Data shown are mean ± standard deviation. Student’s t-test was used to compare the results. n. s., non-significant; ***p < 0.001.

To determine whether 4-MC alleviates HG + PA-induced cardiomyocyte apoptosis via the PI3K-AKT signaling pathway, we inhibited the PI3K-AKT pathway using LY294002 (A broad-spectrum PI3K inhibitor) (Li et al., 2023). The addition of LY294002 blocked the restorative effect of 4-MC on HG + PA-induced cardiomyocyte apoptosis (Figures 7A,B). Western blot results demonstrated that, after the addition of LY294002, the expression patterns of Bax, Bcl-2, and cleaved Caspase-3 were similar to those in the HG and PA co-treatment group and significantly different from those in the 4-MC treatment group (Figures 7C–E). The In-Cell Western results were consistent with these findings (Figures 7F–H). Finally, the CCK-8 assay likewise demonstrated that fulvestrant and LY294002 can abrogate the protective effect of 4-MC against HG and PA co-treatment–induced cardiomyocyte death (Figure 7I). Taken together, 4-MC activates ESR1 to relieve the suppression of the PI3K–AKT signaling pathway caused by high glucose and high fat, thereby reducing oxidative stress in diabetic cardiomyocytes and inhibiting cardiomyocyte apoptosis.

**FIGURE 8:**
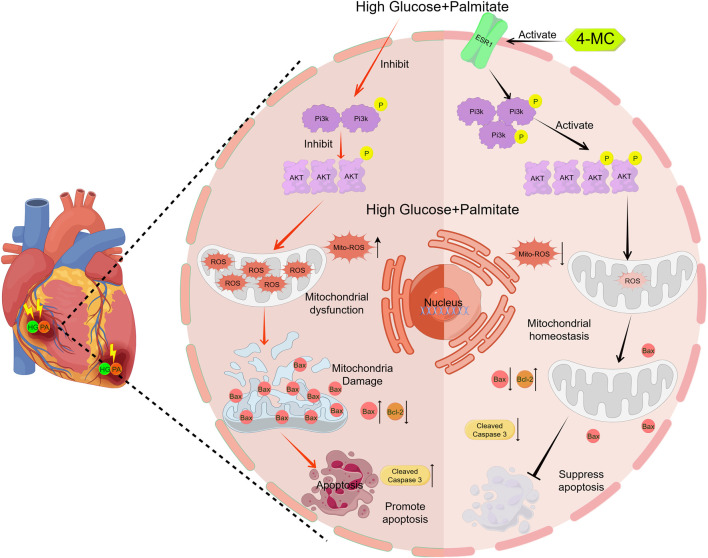
4-MC protects cardiomyocytes against high glucose/high lipid-induced oxidative stress and apoptosis by targeting ESR1-mediated activation of the PI3K-Akt signaling pathway. In AC16 cardiomyocytes, high glucose and high lipid suppress the PI3K-Akt signaling pathway, leading to mitochondrial oxidative stress, ROS generation, and subsequent apoptosis. Conversely, 4-methylcatechol (4-MC) targets ESR1 to reactivate PI3K-Akt signaling, thereby alleviating mitochondrial oxidative stress and preventing apoptosis.

## 4 Discussion

This study demonstrates the protective effects of 4-Methylcatechol (4-MC) against diabetic myocardial disorder. Diabetic myocardial disorder is a serious complication of diabetes, wherein high glucose and high lipid levels play a critical role in its pathogenesis. Developing targeted therapeutic agents for diabetic myocardial disorder is of paramount importance. In this study, we utilized an AC16 cardiomyocyte injury model induced by high glucose (HG, 35 mM) and palmitic acid (PA, 100 μM) to investigate the cardioprotective effects of 4-MC against HG + PA-induced cardiomyocyte injury and the underlying mechanisms.

In recent years, multiple distinct mechanisms have been proposed to contribute to the pathogenesis of diabetic myocardial disorder, primarily including oxidative stress, cardiomyocyte death, and alterations in mitochondrial function (Ritchie and Abel, 2020; Jia et al., 2018; Wang et al., 2025). Utilizing cellular models, we have conducted in-depth investigations into the pathogenic mechanisms of diabetic myocardial disorder and evaluated the therapeutic potential of 4-MC. In our cellular model, AC16 cardiomyocytes were treated with high glucose (35 mM) combined with palmitic acid (100 μM), successfully modeling the pathological state of diabetic myocardial disorder. This model demonstrated characteristic cellular injury features, including reduced cell viability, decreased mitochondrial membrane potential, and increased apoptosis levels, thereby fully validating the efficacy of this induction protocol.

4-Methylcatechol (4-MC), a key metabolite of quercetin *in vivo*, naturally occurs in various fruits and (Guo et al., 2021; Feliciano et al., 2016). It is formed through intestinal metabolic transformation following quercetin ingestion in humans. Substantial evidence has documented the beneficial effects of 4-MC on human health, including cardioprotection, promotion of osteoclastogenesis, suppression of carcinogenesis, and prevention of acute kidney injury (Paclíková et al., 2025). Furthermore, quercetin, a natural polyphenol, demonstrates multiple biological activities. Studies have revealed its benefits in mental health (anti-anxiety and antidepressant effects), neuroprotection (e.g., against Alzheimer’s disease) (Ahmed et al., 2021; Chen et al., 2021), cardiovascular protection (blood pressure reduction), and skin photoprotection against UV radiation through antioxidant and anti-inflammatory mechanisms. Additionally, it exhibits antiviral and anticancer activities, inhibiting tumor growth via modulation of multiple cellular pathways (Dabeek and Marra, 2019; Xu et al., 2019; Payton et al., 2011). It is noteworthy that 4-methylcatechol (4-MC) exhibits certain forestomach carcinogenic effects in F344 rats (Asakawa et al., 1994), which constitutes a crucial consideration when evaluating its therapeutic potential for both anticancer applications and cardiovascular disease treatment. In summary, combined with 4-MC’s cardioprotective effects and quercetin’s antioxidative and anti-inflammatory properties, this collective evidence strongly supports the significant antioxidant activity and cardiac function preservation capabilities of these compounds (Manville et al., 2025).

Through systematic screening of PubMed and Web of Science databases, we identified 1,951 target genes associated with diabetic myocardial disorder, successfully establishing a diabetic myocardial disorder -related gene set. By integrating target predictions from TCMSP, GeneCards, SwissTargetPrediction (STP), PharmMapper (PM), and BindingDB databases, we obtained potential binding targets of 4-MC. The intersection between the diabetic myocardial disorder -related gene set and 4-MC binding targets was visualized using Venn diagrams. protein–protein interaction (PPI) analysis of the intersecting gene set was performed via the STRING database, leading to the identification of the top 10 core genes. Subsequently, GO enrichment and KEGG pathway analyses were conducted on the diabetic myocardial disorder -related gene set and 4-MC binding targets. The GO/KEGG enrichment results revealed that pathways involved in sensing and responding to oxidative stress, apoptosis, regulation of cardiomyocyte contraction, and cardiomyocyte relaxation play central roles in 4-MC’s action against diabetic myocardial disorder. Key genes enriched were associated with oxidative stress, energy metabolism, and environmental stress responses. Based on these findings, we hypothesize that the mechanism by which 4-MC treats diabetic myocardial disorder may operate through modulation of oxidative stress, apoptosis, and energy metabolism. Molecular docking was subsequently performed to validate the potential targets of 4-MC.

Molecular docking analysis demonstrated that 4-MC forms stable complexes with multiple targets including ESR1, AR, EPHX2, ALOX5, HDAC6, ACHE, and SERPINE1. Specifically, 4-MC establishes hydrogen bond anchoring with Arg394 and Lys449 residues within the ESR1 binding pocket, accompanied by favorable docking scores. Importantly, ESR1 exhibits strong mechanistic alignment with our enrichment analysis results regarding steroid/hormone binding and ligand-activated transcription factor activity, thereby providing substantial biological relevance for diabetic myocardial disorder investigation. These findings collectively indicate that the therapeutic mechanism of 4-MC against diabetic myocardial disorder is intimately associated with ESR1 modulation.

The estrogen receptor ESR1 emerges as a key therapeutic target in heart failure (Chen et al., 2024; Xie et al., 2024), where it orchestrates cardioprotection by regulating cardiomyocyte apoptosis, inflammation, and oxidative stress. Structurally, ESR1-encoded ERα activates the PI3K/AKT pathway through direct interaction with PI3K’s p85 regulatory subunit (Lee et al., 2005). This signaling cascade critically modulates cardiac homeostasis by controlling myocyte survival, vascular remodeling, and inflammatory responses, while also mediating cardiotoxicity induced by diverse insults including LPS, oxidative stress (H2O2), heavy metals (mercury), and chemotherapeutic agents (doxorubicin) (Ghafouri-Fard et al., 2022; Zhang et al., 2022). Based on ESR1’s cardiovascular protective role and computational evidence of 4-MC binding to ESR1-PI3K-AKT axis, we propose that 4-MC exerts therapeutic effects against dilated cardiomyopathy through this molecular pathway.

Subsequently, using an *in vitro* diabetic myocardial disorder model, we demonstrated that 4-MC alleviates oxidative stress and endogenous apoptosis in HG/PA-induced cardiomyocytes. The observation that Fulvestrant blocks this protective effect confirms the ESSENTIAL role of ESR1 in mediating 4-MC’s cardioprotection. To further investigate the mechanism underlying 4-MC’s ESR1-mediated protection against HG/PA-induced injury, we utilized the PI3K inhibitor LY294002, which similarly abolished 4-MC’s protective effects. These results validate that 4-MC alleviates HG/PA-induced cardiomyocyte injury through the ESR1-PI3K-AKT pathway.

It should be noted that the current study lacks *in vitro* binding assays to evaluate the interaction between 4-MC and ESR1, and the entire signaling pathway awaits further validation through *in vivo* experiments. However, based on bioinformatics analyses including molecular dynamics simulations and molecular docking, we have confirmed the stable binding between 4-MC and ESR1. In future work, we will employ complementary strategies such as surface plasmon resonance (SPR), isothermal titration calorimetry (ITC), and kinase activity assays to quantitatively characterize the binding affinity, kinetic parameters, and functional effects of the 4-MC-ESR1 interaction. Furthermore, the critical role of ESR1 in mediating the effects of 4-MC requires definitive genetic evidence. Currently, it remains unclear whether the protective effect of 4-MC on the pathway depends on this receptor in ESR1-knockout models. *In vivo* studies will be conducted to further elucidate how 4-MC alleviates high glucose/palmitic acid (HG/PA)-induced cardiomyocyte injury through ESR1-mediated PI3K-AKT pathway activation.

## 5 Conclusion

In summary, This study demonstrates that 4-methylcatechol (4-MC), a major bioactive metabolite of quercetin, alleviates high glucose and palmitic acid-induced cardiomyocyte oxidative stress and apoptosis through estrogen receptor alpha (ESR1)-mediated activation of the PI3K-AKT signaling pathway. Molecular docking and molecular dynamics simulations revealed stable binding between 4-MC and ESR1, characterized by hydrogen bond anchors at Arg394 and Lys449 and a favorable docking score (−5.8 kcal/mol). Pharmacological blockade with the ESR1-specific antagonist Fulvestrant confirmed the functional dependence of 4-MC’s cardioprotective effects on this receptor. These findings uncover a novel mechanism of action for 4-MC, identify ESR1 as a direct molecular target, and establish a previously unrecognized link between 4-MC, ESR1, and cardiac protection in diabetic myocardial disorder.

## Data Availability

The datasets presented in this study can be found in online repositories. The names of the repository/repositories and accession number(s) can be found in the article/Supplementary Material.
