# Supplementary material for: 4-Methylcatechol attenuates diabetic myocardial disorder via the ESR1–PI3K–AKT pathway
Source: Front Pharmacol. 2026 Apr 9;17:1801465. doi: 10.3389/fphar.2026.1801465 (PMC13102569; doi:10.3389/fphar.2026.1801465)
Supplement: Supplementary file 1 [file Supplementaryfile1.docx]

Supplementary Material

## Supplementary Figures


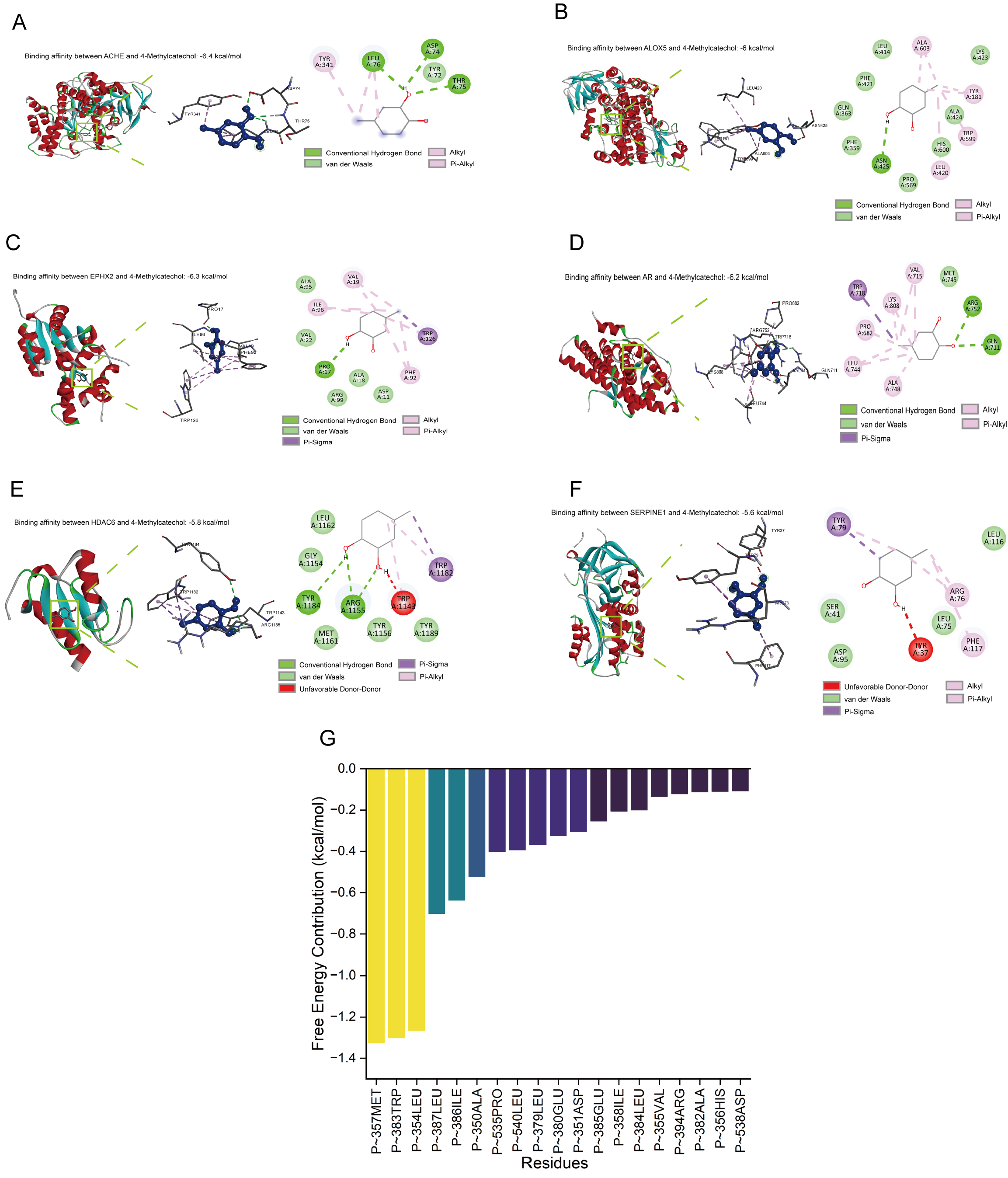


Figure. S1. Molecular docking(A) 4-MC bound to ACHE (ΔG=−6.4kcal/mol). (B) 4-MC bound to ALOX5(ΔG=−6.0kcal/mol). (C) 4-MC bound to EPHX2 (ΔG=−6.3kcal/mol) (D) 4-MC bound to AR (ΔG=−6.2kcal/mol). (E) 4-MC bound to HADC6 (ΔG=−5.8kcal/mol). (F) 4-MC bound to SERPINE1 (ΔG=−5.6kcal/mol). (G) Per-residue free-energy contributions.


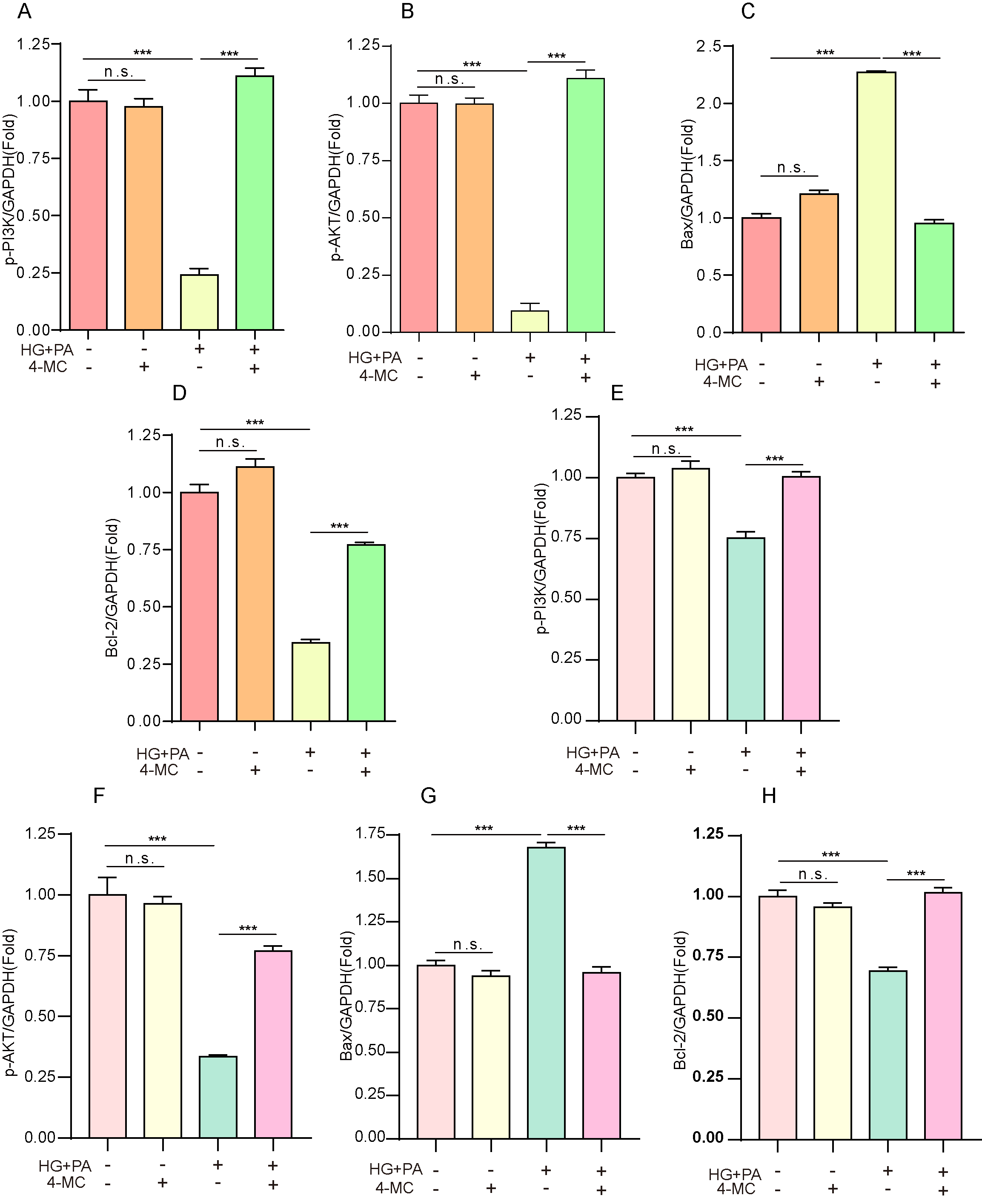


Figure. S2. Western Blot Quantification of p-PI3K,p-AKT,Bax and Bcl-2 Protein Expression.(A-B) Western blot analysis of p-PI3K/GAPDH and p-AKT/ GAPDH expression patterns. (C-D) Western blot analysis of Bax/GAPDH and Bcl-2/ GAPDH expression patterns. (E-F) In-Cell Western analysis of p-PI3K/GAPDH and p-AKT/ GAPDH expression patterns. (G-H) In-Cell Western analysis of Bax/GAPDH and Bcl-2/ GAPDH expression patterns. Data shown are mean ± standard deviation. Student's t-test was used to compare the results. n.s.,non-significant; ***p<0.001.


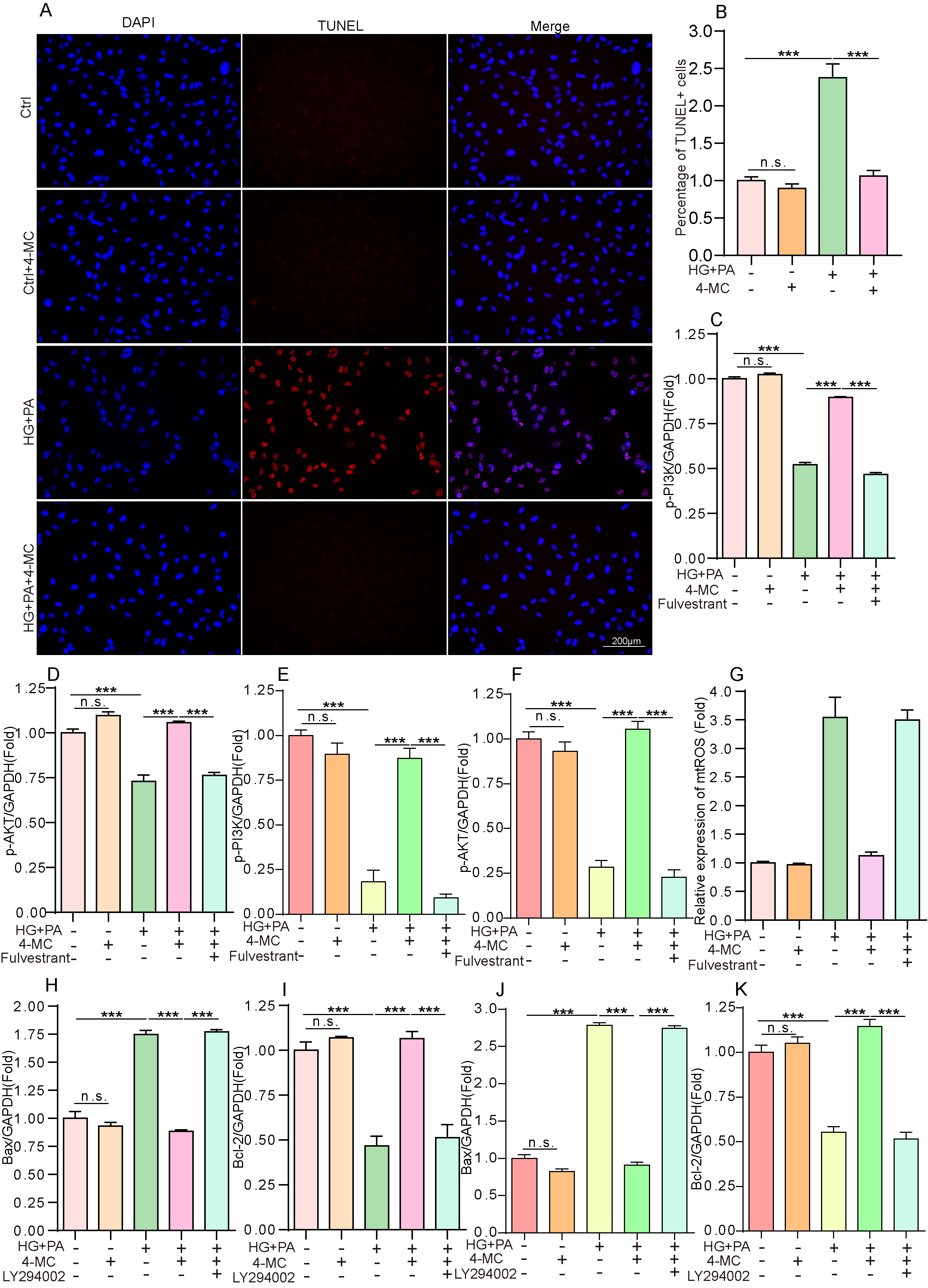


Figure. S3. 4-MCattenuates HG+PA induced oxidative stress and apoptosis via ERS1-mediated activation of PI3K/AKT signaling pathway (A-B) TUNEL assay detects apoptosis in AC16 cells. (C-F) Western blot and In-Cell Western analysis of p-PI3K/GAPDH and p-AKT/ GAPDH expression patterns. (G) Quantitative analysis of mitochondrial superoxide levels by MitoSOX Red staining. (H-K) In-Cell Western andWestern blot analysis of Bax/GAPDH and Bcl-2/ GAPDH expression patterns. Data shown are mean ± standard deviation. Student's t-test was used to compare the results. n.s.,non-significant; ***p<0.001.
